# Visual Attention to Alcohol Cues and Responsible Drinking Statements Within Alcohol Advertisements and Public Health Campaigns: Relationships With Drinking Intentions and Alcohol Consumption in the Laboratory

**DOI:** 10.1037/adb0000284

**Published:** 2017-05-11

**Authors:** Inge Kersbergen, Matt Field

**Affiliations:** 1 Department of Psychological Sciences, University of Liverpool, and UK Centre for Tobacco and Alcohol Studies (UKCTAS), Liverpool, United Kingdom

**Keywords:** advertising, alcohol, drinking intentions, public health campaigns, visual attention

## Abstract

Both alcohol advertising and public health campaigns increase alcohol consumption in the short term, and this may be attributable to attentional capture by alcohol-related cues in both types of media. The present studies investigated the association between (a) visual attention to alcohol cues and responsible drinking statements in alcohol advertising and public health campaigns, and (b) next-week drinking intentions (Study 1) and drinking behavior in the lab (Study 2). In Study 1, 90 male participants viewed 1 of 3 TV alcohol adverts (conventional advert; advert that emphasized responsible drinking; or public health campaign; between-subjects manipulation) while their visual attention to alcohol cues and responsible drinking statements was recorded, before reporting their drinking intentions. Study 2 used a within-subjects design in which 62 participants (27% male) viewed alcohol and soda advertisements while their attention to alcohol/soda cues and responsible drinking statements was recorded, before completing a bogus taste test with different alcoholic and nonalcoholic drinks. In both studies, alcohol cues attracted more attention than responsible drinking statements, except when viewing a public health TV campaign. Attention to responsible drinking statements was not associated with intentions to drink alcohol over the next week (Study 1) or alcohol consumption in the lab (Study 2). However, attention to alcohol portrayal cues within alcohol advertisements was associated with ad lib alcohol consumption in Study 2, although attention to other types of alcohol cues (brand logos, glassware, and packaging) was not associated. Future studies should investigate how responsible drinking statements might be improved to attract more attention.

Alcohol is widely advertised, and exposure to advertising increases drinking behavior. For example, in 2012 there were on average 1.24 alcohol references per minute in TV broadcasts of European championship football matches (Adams, Coleman, & White, 2014), and a recent ecological momentary assessment study showed that young adolescents in the U.S.A. are exposed to an average of 2.7 alcohol advertisements per day (Collins et al., 2016). Exposure to alcohol advertising affects drinking behavior in both the short and the long term. A recent meta-analysis revealed a robust (albeit small) effect of exposure to alcohol advertisements on immediate alcohol consumption among adults (SMD = 0.20, 95% CI = 0.05, 0.34; Stautz, Brown, King, Shemilt, & Marteau, 2016). In the long term, the effect of alcohol advertising on drinking behavior in adolescents is dose dependent: Greater exposure to alcohol advertisements over time predicts earlier onset of drinking and increased quantity of alcohol consumed (Anderson, de Bruijn, Angus, Gordon, & Hastings, 2009; L. A. Smith & Foxcroft, 2009). To the best of our knowledge, the long-term effect of alcohol advertising on alcohol consumption in adults has not been investigated.

In an attempt to counter the effects of alcohol advertising and other forms of marketing, alcohol public health campaigns and responsible drinking statements within alcohol advertising are commonly used by governments to reduce alcohol-related harm and improve public health (e.g., “Change4Life”; Public Health England, 2012). In the United Kingdom, TV alcohol adverts voluntarily incorporate a responsible drinking statement to promote drinkaware.co.uk, an industry-funded website that gives “comprehensive advice to the public on responsible drinking” (Portman Group, n.d.). As part of the “responsibility deal” (Department of Health, 2011), a link to the Drinkaware website should be displayed on all alcohol marketing (e.g., print, TV, and online adverts) and on alcohol packaging. The inclusion of responsible drinking statements is encouraged, but not mandatory. In order to comply with the voluntary agreement, the website link (and any additional responsible drinking statements) on TV adverts must be displayed for a minimum of four seconds and include the words “*For the facts* [about alcohol]; drinkaware.co.uk” (“Drinkaware Brand Guidelines For Partners,” 2009).

Research on the effectiveness of alcohol public health campaigns and responsible drinking statements embedded in alcohol marketing is mixed. Stautz and Marteau (2016) demonstrated that viewing TV alcohol public health campaigns reduced the urge to drink, compared to alcohol promoting adverts and neutral adverts, in young adults. Increased negative affect after watching the public health campaigns mediated this effect. However, other researchers observed limited or no effect of public health campaigns or responsible drinking statements on drinking behavior (see Agostinelli & Grube, 2002 for a review), or even unanticipated effects, such as increased alcohol consumption (Moss et al., 2015) or reduced negative attitudes toward alcohol (Brown, Stautz, Hollands, Winpenny, & Marteau, 2016). Some researchers suggest that the limited effectiveness of responsible drinking statements might be attributed to their design and content, as they generally provide little information about alcohol-related harms and provide no clear goals for behavior change (Al-hamdani, 2014; Martin-Moreno et al., 2013; Wilkinson & Room, 2009).

Individual differences in attentional biases for alcohol-related cues (i.e., the tendency to preferentially direct attention toward those cues) may partially explain why alcohol advertisements and public health campaigns do not consistently influence drinking behavior. In a recent theoretical model, Field et al. (2016) argued that attentional bias fluctuates in line with the underlying motivational state, and the bias exerts a causal influence on proximal, but not distal, drinking behavior. On this basis, we suggest that individual differences in attention to different types of visual cues and text statements within alcohol advertising should mediate the influence of those cues/statements on alcohol consumption that occurs soon afterward. Specifically, attention to responsible drinking statements should be negatively correlated, and attention to alcohol-related cues positively correlated, with alcohol consumption and intentions to drink measured immediately afterward. Relevant here is a recent study (Moss et al., 2015) in which participants were exposed to either responsible drinking posters (Drinkaware) or general public health posters (Change4Life), while their attention was monitored with an eye tracker. Immediately after viewing posters, their ad libitum alcohol consumption was measured with a bogus “taste test.” Results indicated that participants who viewed Drinkaware posters attended to images that depicted the positive consequences of alcohol consumption for longer than images that depicted the negative consequences of alcohol consumption and responsible drinking statements. Participants who viewed Drinkaware posters also consumed more alcohol during the taste test than participants who viewed Change4Life posters. The authors suggested that individual differences in allocation of attention to alcohol cues may have accounted for the observed group differences in alcohol consumption, but they did not test this formally.

The purpose of the current studies was to assess visual attention to alcohol cues and responsible drinking statements in alcohol advertising and public health campaigns, and investigate how individual differences in attention predict intentions to drink (Study 1) and drinking behavior in the lab (Study 2). A secondary aim was to gather descriptive information about how much attention people typically direct to responsible drinking statements in public health campaigns and conventional TV alcohol advertisements, because this information is not currently available.

## Study 1

The purpose of this study was to measure alcohol consumers’ visual attention to alcohol cues and responsible drinking statements in TV alcohol adverts and public health campaigns, and investigate how this predicts drinking intentions. In a previous study, responsible drinking statements captured more attention when they were presented in alcohol advertisements that emphasized responsible drinking compared to when they were presented in conventional alcohol promoting advertisements (Thomsen & Fulton, 2007). Therefore, the context in which responsible drinking statements are communicated might be an important moderator of the effectiveness of those statements. Responsible drinking statements can either be embedded in alcohol marketing or communicated independently (i.e., public health campaigns). It has been argued that responsibility statements in alcohol marketing are predominantly used as an additional means to promote the product rather than convey public health information (K. C. Smith, Cukier, & Jernigan, 2014). K. C. Smith et al. (2014) showed a variety of strategies that the alcohol industry uses to utilize responsibility statements as a marketing tool, such as using responsibility statements to make promises about the product’s effect (e.g., “enjoy responsibly”). This seems to be a successful strategy, as public health campaigns sponsored by individual alcohol brands have been shown to maintain and even increase positive brand evaluations (S. W. Smith, Atkin, & Roznowski, 2006). A parallel literature on food advertising showed that an advert for ”healthy” fast food meals did not increase healthier food choices in children, but did increase liking for fast food in general (Boyland, Kavanagh-Safran, & Halford, 2015).

In the present study, we contrasted participants’ visual attention to alcohol cues and responsible drinking statements in alcohol adverts and a public health campaign, and investigated how viewing patterns predicted subsequent drinking intentions. Participants were exposed to one of three short videos while we measured their eye movements: a conventional alcohol public health campaign from Drinkaware; a Heineken alcohol advert with a clear emphasis on responsible drinking; or a conventional Heineken alcohol advert. Regarding participants’ eye movements, based on Thomsen and Fulton (2007), we hypothesized that participants who viewed either Heineken advert would attend more to alcohol cues than responsible drinking statements, but the opposite would be the case for participants who viewed the Drinkaware advert. We also hypothesized that participants who viewed the Drinkaware advert would pay more attention to responsible drinking statements than participants who viewed either of the Heineken adverts.

Regarding participants’ drinking intentions, we selected this as an outcome measure on the basis of findings from a recent study that demonstrated that a single exposure to an antibinge drinking campaign affected students’ intentions to refrain from binge drinking in the subsequent two weeks (Hendriks, De Bruijn, & van Den Putte, 2012), and also because Drinkaware (who commissioned the public health campaign used in this study) aims to “raise awareness of alcohol and its harm” (Drinkaware, 2016) and therefore it is likely that the current video was designed with that aim in mind. We hypothesized that participants who watched the Drinkaware advert would intend to drink less alcohol in the subsequent week compared to those exposed to the conventional Heineken advert and the Heineken advert with a responsible drinking message, but drinking intentions would not differ across participants who viewed the two different Heineken adverts. Regarding hypothesized interrelationships between attention and drinking intentions, based on Moss et al. (2015), we hypothesized that attention to the responsible drinking statements would be negatively correlated with the amount of alcohol that participants intended to drink in the near future, whereas attention to the alcohol cues would be positively correlated with intended alcohol consumption.

### Method

#### Participants

We recruited 90 participants to take part in this study, which had a between-subjects design. Participants had to be male and at least 18 years old. We recruited males only, as the lead characters in the adverts and public health campaign that we presented were all male and therefore we considered men to be the target audience for the adverts (see description of advert content, below). In order to capture participants with a range of drinking behaviors, regular alcohol consumption was not an eligibility criterion. However, three participants were abstainers and were subsequently excluded from all analyses. See Table 1 for participant characteristics. The study received ethical approval from the University of Liverpool Ethics Committee. Testing took place between October 2015 and July 2016.[Table-anchor tbl1]

#### Advertising/public health campaign manipulation

Participants viewed five videos: four neutral adverts (e.g., comparison websites, insurance), and one of three target adverts/public health campaigns (conventional Heineken advert, Heineken advert with responsible drinking message, or Drinkaware; hereafter referred to as “target videos”). The videos were displayed in the same order in each condition, with the target video always being displayed as the fourth advert of five. The target video was varied on a between-subjects basis, but the neutral adverts were the same for all participants. We monitored participants’ eye movements while they viewed the adverts using a Gazepoint GP3 eye-tracker sampling at 60Hz (Gazepoint, Vancouver, Canada).

##### Drinkaware (“Drink Less Miss Less (feat. Lauren Laverne),” 2009; 37 seconds)

This public health campaign shows a Lauren Laverne gig at an outdoor music festival. She asks the crowd if they are enjoying themselves and if she should join them and crowd surf. The audience is shown drinking beer and cheering her on. After she jumps into the crowd, she falls into an empty patch of grass. Then we see a crowd of men and women gathered around a tree. Some are urinating against the tree and others are waiting in a queue. Then, the following text was displayed (and spoken): “Alcohol makes you pee more than water or soft drinks—pace yourself and miss less,” followed by a figure showing the UK government guidelines for lower-risk alcohol consumption. The advert ended with the displayed text “Drink less, miss less” and the drinkaware.co.uk logo. The advert was aired in the UK in 2009 (“Drink Less Miss Less,” 2009).

##### Heineken advert with responsible drinking message (“Heineken ‘Dance More Drink Slow’ campaign,” 2016; 60 seconds)

This advert shows several snapshots, at different time stamps, of a night out in a club. The first time stamp is at 11.45 p.m. and the last is at 6.12am. The main character in this advert is a young male who is on a night out in this club. He starts his night out by ordering and drinking a bottle of Heineken beer. The next time that he orders a drink, he refuses a bottle of Heineken and requests water instead. As the night progresses, people around him get more drunk and get into embarrassing situations (e.g., falling over). Throughout the advert, there is a girl who, like the main character, also drinks water and stays sober. At the end of the night, they lock eyes and walk out of the club holding hands. Then, the following text is displayed: “Enjoy the sunrise. Dance more - Drink slow”, followed by the Heineken logo. There was no dialogue and there was club music playing in the background. The advert was aired in the UK in 2014 (Heineken, 2014).

##### Heineken (traditional advert; “Heineken The Date,” 2011; 91 seconds)

This advert showed a man and a woman on a date. They enter a restaurant/theater via a secret entrance, followed by a series of brief high-energy encounters between the duo and other characters (e.g., kitchen staff, waiters, other guests). The pair ends at a table, clinking Heineken bottles. There was no dialogue and there was Bollywood music playing in the background. The advert aired in the UK in 2012 (Horsnell, 2012).

#### Drinking intentions

We measured drinking intentions with three different measures: Next week drinking intentions, next week binge drinking intentions, and drinking intentions for the next drinking occasion.

##### Next week (Glock & Krolak-Schwerdt, 2013)

We asked participants whether they intended to drink alcohol in the next week (yes/no). If participants answered yes, we asked how many pints of beer/cider, 175 ml glasses of wine, and shots of spirits they intended to drink in the next week. We calculated their intended consumption in UK units based on their answers (2 UK units for a pint of beer/cider or a 175 ml glass of wine and 1 UK unit for a shot of spirits—units were based on the SIPS brief intervention tool; Kaner et al., 2013).

##### Next week binge drinking (Elliott & Ainsworth, 2012)

We measured next week binge drinking intentions with three questions (“Do you intend to binge drink next week?” “To what extent do you intend to binge drink next week?” “How much do you want to engage in a binge-drinking session in the next week?”). Participants responded to each item on a 9-point Likert scale with anchors 1 = *definitely yes/not at all/not at all*, 9 = *definitely no/great extent/a lot*, respectively. Answers were recoded so that higher values represented greater intention to binge drink and were averaged into a single binge drinking intentions score (α = .90).

##### Next drinking occasion

We used a hypothetical menu task, based on Boyland et al. (2015), to measure how many units of alcohol participants intended to consume on their next drinking occasion. We asked participants to imagine their next drinking occasion and consider what and how much they wanted to drink. They were shown a bar menu with >100 alcoholic and nonalcoholic drinks. We asked participants to imagine that the drinks on the menu were the only drinks on offer during their next drinking occasion, regardless of what venue they were in. They were instructed to indicate which drinks they would like to consume. After selecting their drink choices, they were asked to specify how many drinks of each type they would consume. They were specifically instructed to only consider drinks they would consume themselves (even if someone else would pay for them) and to disregard anything they might purchase for other people. To corroborate the cover story, the prices were blacked out. We calculated how many units of alcohol participants intended to consume based on the ABV of the drinks they selected.

#### Procedure

Participants were recruited to take part in a study investigating advertising and price receptivity. They were told that they would view some advertisements, followed by a hypothetical purchasing task (the “next drinking occasion” measure of drinking intentions, as described above). They were informed that some participants would see the prices of the products during the task, whereas others would not. In reality, no one saw any product prices throughout the experiment. After arrival in the lab, participants were randomly allocated to one of three experimental conditions. Participants were asked to view the five videos, followed by a bogus measure of product choice relating to one of the neutral adverts (this was to corroborate the cover story) and the three measures of drinking intentions. While viewing the videos, we monitored participants’ eye movements using a Gazepoint GP3 eye-tracker (Gazepoint, Vancouver, Canada). We measured how long (in seconds) participants fixated on alcohol cues and responsibility statements in the target video. Then, they filled out a set of questionnaires measuring recent alcohol consumption (a 14-day retrospective timeline followback diary; Sobell & Sobell, 1992); hazardous drinking (Alcohol Use Disorders Identification Test **[**AUDIT**]**; Saunders, Aasland, Babor, de la Fuente, & Grant, 1993); motivation to reduce drinking (Temptation and Restraint Inventory **[**TRI**]**; Collins & Lapp, 1992; Readiness to Change Questionnaire **[**RTCQ**]**; Rollnick, Heather, Gold, & Hall, 1992; and contemplation ladder; LaBrie, Quinlan, Schiffman, & Earleywine, 2005); and craving (Approach and Avoidance of Alcohol Questionnaire—Right now version **[**AAAQ**]**; McEvoy, Stritzke, French, Lang, & Ketterman, 2004). A motivation to reduce drinking score was created by averaging the TRI restraint subscale, the RTCQ contemplation and action subscales, and the contemplation ladder as these scales were strongly correlated (*r* = .48–.62, *p*s < .001, α = .81). Then, we asked participants what they thought the aims of the study were and whether they had seen the target video prior to the experiment. Finally, they were thanked and debriefed. The study took 15–20 min and participants were reimbursed with a £5 shopping voucher or partial course credits.

##### Processing of eye tracking data

We analyzed participants’ visual attention to all alcohol cues and responsible drinking statements displayed during the target videos. Alcohol cues were defined as all occasions that an alcohol product was consumed or displayed in a glass or in packaging, and as any displays of the Heineken brand logo (there were no brand logos in the Drinkaware video). Responsible drinking statements were defined as the link to drinkaware.co.uk and any additional text that prompted people to reduce their alcohol consumption. Only the Heineken responsibility and Drinkaware videos displayed responsible drinking statements (Heineken: “Dance more, drink slow”; Drinkaware: “Alcohol makes you pee more than water or soft drinks—pace yourself and miss less,” “Daily guidelines: Men: 3–4 units, Women: 2–3 units,” “Drink less, miss less. Drinkaware.co.uk/missless,” “For the facts about alcohol; drinkaware.co.uk”). The size and display duration of alcohol cues and responsible drinking statements varied between target videos; see Table 2 and Figure S1. Therefore, we analyzed visual attention using gaze duration as a proportion of total cue display duration in each particular advert.[Table-anchor tbl2]

Prior research suggests that different types of alcohol cues (complex vs. simple; social vs. nonsocial) vary in the extent to which they capture the attention of alcohol consumers (Forestell, Dickter, & Young, 2012; Miller & Fillmore, 2010). In addition to generic alcohol marketing cues (brand logos, product placement), attention to the portrayal of alcohol consumption in alcohol advertising (i.e., an actor consuming an alcoholic beverage) might be a particularly important predictor of subsequent alcohol consumption, because a previous study demonstrated that participants were likely to sip alcohol in close temporal proximity to an actor sipping alcohol in a movie (Koordeman, Kuntsche, Anschutz, van Baaren, & Engels, 2011). Therefore, we conducted additional exploratory analyses to investigate if attention to specific types of alcohol cues would predict drinking intentions. Alcohol cues were categorized as those depicting: (a) Portrayal: occasions where a person taking a sip of the advertised product was displayed on screen; (b) Packaging: occasions where a branded bottle or can of the advertised product was displayed (excluding occasions that fit under Portrayal); (c) Glass: occasions where the advertised product was displayed in a glass (excluding occasions that fit under Portrayal); and (d) Logo: occasions where the brand logo was displayed separately from the product. See supplementary materials for more information on the coding of the alcohol cues.

### Results

#### Participant characteristics

A multivariate analysis of variance with target video condition as a between-subjects factor and age, recent alcohol consumption, AUDIT scores and motivation to reduce drinking as dependent variables revealed that the multivariate effect of condition was not statistically significant, *F*(8, 162) = 1.49, *p* = .16, η_p_^2^ = .07. Therefore, groups were well-matched.

#### Effect of target video condition on drinking intentions (see Table 3)

We conducted a one-way multivariate analysis of covariance with target video condition as the between-subjects factor and intended consumption at the next drinking occasion (menu task), intended consumption in the subsequent week, and intentions to binge drink in the subsequent week, as dependent variables, with age, AUDIT scores, weekly alcohol consumption, and motivation to reduce drinking as covariates. The multivariate test revealed no overall effect of condition, *F*(6, 158) = .47, *p* = .83, η_p_^2^ = .02. Inspection of the univariate tests confirmed that target video condition did not significantly affect intended consumption at the next drinking occasion, *F*(2, 80) = .16, *p* = .85, η_p_^2^ = .004, intended consumption in the subsequent week, *F*(2, 80) = 1.16, *p* = .32, η_p_^2^ = .03, or intentions to binge drink during the subsequent week, *F*(2, 80) = .46, *p* = .63, η_p_^2^ = .01.[Table-anchor tbl3]

#### Attention to alcohol cues and responsible drinking statements (see Figure 1)

**Figure 1 fig1:**
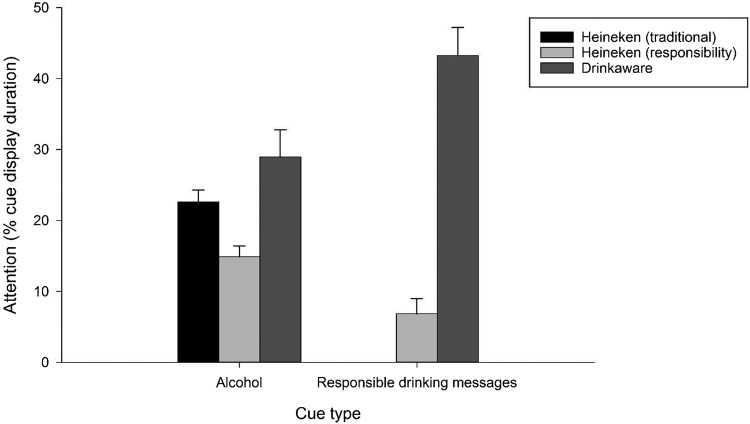
Study 1. Visual attention to alcohol cues and responsible drinking messages in the different advertising conditions. Bars represent gaze duration as a percentage of total cue display time. Error bars indicate SEM. Traditional Heineken advert did not display any responsible drinking messages.

Participants with more invalid fixations (data points with missing data from both eyes) than valid fixations were excluded from analyses due to inaccurate tracking (*n* = 7)^1^. To investigate whether participants in the different target video conditions had different viewing patterns, we conducted a one-way (video condition: Heineken responsibility, Heineken conventional, Drinkaware) analysis of covariance (ANCOVA) with attention to alcohol cues (gaze duration as a percentage of cue display duration) as the DV and age, AUDIT scores, weekly alcohol consumption, and motivation to reduce drinking as covariates. Results showed that attention to alcohol cues significantly differed across target videos, *F*(2, 73) = 7.55, *p* = .001, η_p_^2^ = .17. Post hoc *t* tests showed that participants paid more attention to alcohol cues in the Heineken conventional advert (*M* = 22.61, *SD* = 8.65) than in the Heineken responsibility advert (*M* = 14.88, *SD* = 7.92, *t*(52) = 3.43, *p* = .001, *d* = .95). Participants also paid significantly more attention to alcohol cues in the Drinkaware video (*M* = 28.94, *SD* = 19.12) than in the Heineken responsibility advert, *t*(51) = 3.57, *p* = .001, *d* = 1.00. Participants paid similar amounts of attention to alcohol cues in the Drinkaware video and in the Heineken conventional advert, *t*(49) = 1.53, *p* = .13, *d* = .44.[Fig-anchor fig1]

As the conventional Heineken advert had no responsibility statements, the following analyses were conducted only on the Heineken responsibility advert and the Drinkaware video. A cue type (alcohol, responsible drinking statement) × Target video condition (Heineken responsibility, Drinkaware) repeated-measures ANCOVA with attention as a percentage of cue display time as the DV and age, AUDIT scores, weekly alcohol consumption, and motivation to reduce drinking as covariates showed a nonsignificant main effect of cue type, *F*(1, 48) = 1.86, *p* = .18, η_p_^2^ = .04, and a significant main effect of target video condition, *F*(1, 48) = 35.43, *p* < .001, η_p_^2^ = .43, which were qualified by a significant interaction, *F*(1, 48) = 26.66, *p* < .001, η_p_^2^ = .36. Post hoc *t* tests split by condition showed that participants who viewed the Drinkaware video paid more attention to responsible drinking statements (*M* = 43.26, *SD* = 19.70) than alcohol cues (*M* = 28.94, *SD* = 19.12, *t*(24) = 4.61, *p* < .001, *d* = .92), whereas participants who viewed the Heineken responsibility advert paid more attention to alcohol cues (*M* = 14.88, *SD* = 7.92) than responsible drinking statements (*M* = 6.83, *SD* = 11.30, *t*(27) = 3.16, *p* = .004, *d* = .60).

#### Attention to responsible drinking statements and different types of alcohol cues as correlates of drinking intentions

After controlling for participant characteristics (age, AUDIT scores, weekly alcohol consumption and motivation to reduce drinking), in the sample as a whole there were no significant correlations between attention to alcohol cues (collapsed across conditions) and intended consumption at the next drinking occasion (*r* = .01, *p* = .90), intended consumption in the subsequent week (*r* = −.12, *p* = .30), and intentions to binge drink during the subsequent week (*r* = −.08, *p* = .45). Across the advertisements that displayed responsible drinking statements (Drinkaware, Heineken responsibility; *n* = 53), there were no significant correlations between attention to responsible drinking statements and intended consumption at the next drinking occasion (*r* = .05, *p* = .73), intended consumption in the subsequent week (*r* = −.03, *p* = .83), and intentions to binge drink during the subsequent week, *r* = −.10, *p* = .46.

Then, we investigated whether attention to various alcohol cues and responsible drinking statements were correlated with drinking intentions within the three advertising conditions. As shown in Table S1, there were no significant correlations between attention to alcohol cues or responsible drinking statements and drinking intentions in any of the advertising conditions.

### Discussion

The primary aim of this study was to measure alcohol consumers’ attention to alcohol cues and responsible drinking statements in alcohol-related public health campaigns and alcohol advertising with a focus on responsible drinking, and investigate how this is related to drinking intentions. Our results showed that attention to alcohol cues significantly differed across target videos, but individual differences in attention were not correlated with drinking intentions. Participants who watched the Drinkaware video (alcohol-related public health campaign) and the traditional Heineken advert spent a similar amount of time viewing alcohol cues (proportional to their display duration), and both paid more attention to alcohol cues compared to participants who viewed the Heineken advert with a responsible drinking message. In addition, participants who viewed the Drinkaware video paid more attention to the responsible drinking statements than those who viewed the Heineken responsibility advert. We also found that participants who viewed the Drinkaware advert attended more to the responsible drinking statements than the alcohol cues, whereas the opposite was true for those who viewed the Heineken responsibility advert. All of these differences in attention were roughly proportional to differences in display duration and display size between the videos/adverts. While we controlled for display duration in our analyses, we were not able to control for differences in size. Therefore, our findings are likely to be at least partially attributable to differences between videos/adverts in the visual salience of the alcohol cues/responsible drinking statements that they depict.

In the context of these marked differences between videos/adverts, it is important to note that participants who viewed the Drinkaware video and the traditional Heineken advert did not differ in their attention allocation to alcohol cues, even though alcohol cues were more prominent in the Drinkaware video than in the Heineken advert (this is evident in Table 2). Similarly, participants who viewed the Heineken responsibility advert attended less to alcohol cues than participants who viewed the traditional Heineken advert, even though alcohol cues were similarly prominent in both adverts. The overall picture is that alcohol cues appear to be less “attention grabbing” when they are displayed in a “responsible drinking” context, whereas responsible drinking statements are more “attention grabbing” in this context, but only in a public health campaign rather than a branded advert. Our findings are consistent with Thomsen and Fulton (2007), who demonstrated that responsible drinking statements attracted more attention if they were a prominent part of the advert’s message. Those authors also did not control for the size of components of the advert, so it is possible that alcohol adverts that focused on responsible drinking had larger and more prominent responsible drinking messages, which could have accounted for the increase in attention. In line with K. C. Smith et al. (2014), our finding that alcohol cues attracted more attention than responsible drinking statements in the alcohol advert with a responsible drinking message suggests that the primary aim of this type of advertisement may be to promote the brand rather than encourage responsible drinking. Our findings are not in line with findings reported by Moss et al. (2015), who found that participants paid less attention to responsible drinking statements than positive and negative alcohol imagery in responsible drinking posters. Our findings also do not support their hypothesis that viewing patterns might account for differences in drinking behavior, as we found no significant relationships between visual attention and drinking intentions. However, Moss et al. (2015) did not control for differences in size between the alcohol images and the responsible drinking messages in the posters. The findings from the current study suggest that larger alcohol cues and responsible drinking messages also attracted more attention, so it is possible that size differences might have partially accounted for the findings reported by Moss et al. (2015).

We found no evidence that exposure to public health campaigns or alcohol adverts that emphasize responsible drinking affected participants’ drinking intentions compared to traditional alcohol adverts. At face value, these findings contrast with recent findings from Stautz and Marteau (2016), who demonstrated that participants had a lower urge to drink after watching responsible drinking adverts. However, there are a number of important differences between the studies. In their study, Stautz and Marteau (2016) measured immediate urge to drink (right now), whereas we measured more distal drinking intentions (next week/drinking occasion). Stautz and Marteau also exposed participants to multiple public health campaigns with a variety of themes, whereas we showed participants only one public health campaign. It is likely that different public health campaigns have differential effects on drinking-related outcome measures, which might account for the discrepancy in results. Additionally, a single exposure to an alcohol-related warning message might reduce the urge to drink without being sufficient to influence participants’ intentions to drink; instead, effects on drinking intentions might only emerge after sustained exposure to the warning message. For example, attitudes toward smoking became more negative with increasing exposure to an antitobacco print advert (Reinhard, Schindler, Raabe, Stahlberg, & Messner, 2014). Finally, as we did not include a control condition with a nonalcohol advert, we cannot draw any conclusions about the (in)effectiveness of the specific videos/adverts that were used in the present study.

The aim of Study 2 was to investigate how attention allocation to alcohol cues in alcohol advertising is associated with drinking behavior, as we were not able to investigate this in Study 1. We made a number of methodological changes that enabled us to conduct a test of the hypothesis that attention to responsible drinking statements within alcohol adverts would be negatively correlated with the amount of alcohol that participants consumed in the laboratory, whereas attention to alcohol cues would be positively correlated with alcohol consumption. We changed the alcohol-related outcome measure from drinking intentions to actual alcohol consumption because alcohol advertisements have been shown to increase alcohol consumption immediately after exposure (Stautz et al., 2016). The most important methodological change was the switch from a between-subjects design (in Study 1, participants were exposed to only one type of video/advert) to a within-subjects design in which participants were exposed to a number of different adverts for alcoholic and nonalcoholic drinks. This methodological change meant that we were unable to investigate the causal influence of different types of alcohol adverts versus public health campaigns on alcohol consumption. However, the use of multiple different adverts enabled us to clearly distinguish attention to different types of alcohol cues that were depicted in adverts (portrayal, packaging, glass, logo, responsible drinking statements), and investigate the relationship between attention to each of these components and subsequent drinking behavior.

## Study 2

The purpose of this study was to measure participants’ visual attention to various alcohol cues and responsible drinking statements in alcohol advertising and investigate how this predicts alcohol consumption in the laboratory. Therefore, we conducted a cross-sectional study to investigate the relation between attention to these specific alcohol cues and responsible drinking statements and drinking behavior. To investigate how attention to different types of alcohol cues in TV alcohol advertising predicted subsequent alcohol consumption, we asked participants to complete a bogus taste test shortly after viewing alcohol and soda advertisements. We included soda advertisements to investigate whether the association between attention allocation and alcohol consumption was specific to alcohol-related cues or if it could be explained by increased attention to appetitive drinks-related cues in general. We correlated alcohol consumption during the taste test with attention to subtypes of alcohol and soda cues (portrayal of consumption, packaging, drinks glasses, and brand logos) and responsible drinking statements in alcohol advertising. Our primary hypothesis was that greater attention to alcohol cues would predict greater alcohol consumption, and increased attention to responsible drinking statements would predict reduced consumption. Additionally, our secondary hypothesis was that out of the four different types of alcohol cues, alcohol portrayal would be the strongest predictor of alcohol consumption, on the basis of a previous finding that participants were likely to sip alcohol in close temporal proximity to an actor sipping alcohol in a movie (Koordeman, Kuntsche, et al., 2011). As Study 1 demonstrated that alcohol cues in alcohol advertising attracted more attention than responsible drinking messages, Study 2 also investigated whether this pattern would be consistently seen across multiple adverts.

### Method

#### Participants

Sixty-two participants (73% female) took part in this study, which employed a within-subjects design (see Table 1). Participants were eligible to take part if they were aged over 18, drank at least 10 UK units/week (to capture social alcohol consumers who drank regularly), and liked apple cider and cola (as the experiment involved consuming these drinks). The study received ethical approval from the University of Liverpool Research Ethics Committee. Testing took place between September 2015 and February 2016. Participants could take part in both Study 1 and Study 2 under the condition that the testing sessions were at least seven days apart. Six participants participated in both studies.

#### Cover story

At the start of the study, participants were told the following cover story:

We are interested in how alcohol advertising affects how much we like/dislike the taste of alcoholic drinks. During the experiment you will be asked to view alcohol and soft drinks advertisements, while we measure your eye-movements using an eye-tracking camera. After that you will be asked to taste and rate some drinks that you have seen the advertisement for. One group of participants will be shown the brands of the drinks in the taste test, whereas the other group will not receive this information.

In reality, there was no manipulation and no participants were told which brands were used in the taste test.

#### Eye-tracking task

Participants were asked to view a series of advertisements as if they were watching them in an advert break on TV. During the eye-tracking task, participants viewed 8 alcohol (4 cider, 3 beer, 1 spirits) and 8 soda advertisements. All adverts had been aired between 2012 and 2015. The order of presentation was randomized. Each alcohol advert included a link to the Drinkaware website and an optional responsibility statement (“Drinkaware Brand Guidelines For Partners,” 2009). None of the soda adverts showed a responsibility statement. While viewing the adverts, we monitored participants’ eye movements using a Gazepoint GP3 eye-tracker (Gazepoint, Vancouver, Canada). We measured how long (in seconds) participants fixated on alcohol/soda cues and responsibility statements. As in Study 1, we differentiated between four different types of alcohol (and soda) cues: (1) Portrayal: occasions where a person taking a sip of the advertised product was displayed on screen; (2) Packaging: occasions where a branded bottle or can of the advertised product was displayed (excluding occasions that fit under Portrayal); (3) Glass: occasions where the advertised product was displayed in a glass (excluding occasions that fit under Portrayal); and (4) Logo: occasions where the brand logo was displayed separately from the product. As in Study 1, cues varied considerably in display duration (see Figure S2). To control for the variance in display duration, attention to each type of cue was defined as a percentage of total cue display duration in the different advert types (alcohol, soda).

#### Taste test

Ad libitum alcohol consumption was measured under the guise of a taste test (Jones et al., 2016). Participants were given 2 glasses of Bulmers apple cider (440 ml total) and 2 glasses of Pepsi cola (440 ml total). The glasses were marked with numbers 1 to 4, and participants were not informed what brand was contained in each glass. They were asked to taste and rate each drink on eight attributes (e.g., smoothness, sweetness). Each participant was given exactly 10 min to complete this task, after which the experimenter measured how much liquid was left in each glass. An alcohol consumption score was created by dividing cider consumption by total consumption (cider + cola consumption), resulting in a measure of alcohol consumption as a percentage of total volume consumed.

#### Procedure

After providing informed consent, participants completed the eye-tracking task, followed by the taste test. After this, participants completed the same questionnaire battery as administered in Study 1. As in Study 1, a motivation to reduce drinking score was created by averaging the TRI restraint subscale, the RTCQ contemplation and action subscales, and the contemplation ladder (because these scales were highly correlated, *r* = .53–.80, *p*s < .001). We measured familiarity with the Drinkaware website with a single multiple choice question that asked which URL was displayed in each advert (options: drinkaware.co.uk, alcoholfacts.co.uk, alcoholaware.co.uk, drinkfacts.co.uk; displayed in a random order). We also asked whether participants were aware of the website before the study, whether they had ever visited the website, and, if so, how much they liked it (100 mm VAS Scale). Additionally, we used a single multiple choice question to ask about the content on drinkaware.co.uk (options: “Information about alcohol units,” “Advertising for different alcohol brands,” “Tips on reducing your drinking,” “Cocktail recipes,” displayed in a random order; participants were instructed to select all that apply). At the end of the study, we asked participants to write down what they thought the aims of the study were and whether they thought the real purpose of the taste test was to measure their alcohol consumption (yes/no). Finally, participants were thanked and debriefed. Participants received study credits or a £5 shopping voucher.

### Results

#### Viewing patterns (see Figure 2)

**Figure 2 fig2:**
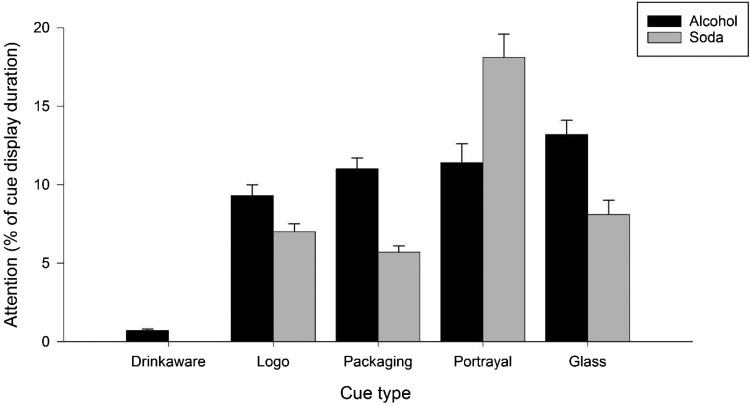
Viewing patterns for alcohol and soda advertisements, split by attention to the Drinkaware (alcohol adverts only), Brand, Packaging, Portrayal, and Glass cues. Bars represent gaze duration as a percentage of total cue display time. Error bars indicate SEM.

Participants with more invalid fixations than valid fixations were excluded from analyses due to inaccurate tracking (*n* = 4)^2^. Participants spent 0.19 s (*SD* = .05) in total looking at the responsible drinking statements over the course of the 8 alcohol advertisements^3^ (*M* = .02 s per advert, *SD* = .04), which is equivalent to 0.65% of the total amount of time that the statements were displayed (total display time = 29.01 s; *M* = 3.63 s per advert, *SD* = 1.29). A one-way (cue/statement type: responsible drinking statements, portrayal cues, packaging cues, glass cues, logo cues) repeated measures analysis of variance (ANOVA) revealed a significant main effect on attention (as a proportion of cue display time; *F*(4, 54) = 63.79, *p* < .001, η_p_^2^ = .83). Bonferroni corrected post hoc comparisons showed that participants paid significantly less attention to the responsibility statements than any of the alcohol cues (all *p*s < .001). Additionally, attention to alcohol portrayal cues did not differ significantly from attention to any other alcohol cue (all *p*s > .38). All other comparisons between alcohol cues were significant (all *p*s < .004).[Fig-anchor fig2]

A drink type (alcohol, soda) × Cue Type (portrayal, packaging, glass, logo) repeated measures ANOVA was conducted to compare viewing patterns of brand-related cues between alcohol and soda advertisements. This revealed a significant main effect of drink type, *F*(1, 57) = 13.72, *p* < .001, η_p_^2^ = .19, and cue type, *F*(3, 55) = 20.33, *p* < .001, η_p_^2^ = .53, which were qualified by a significant Drink Type × Cue Type interaction, *F*(3, 55) = 26.07, *p* < .001, η_p_^2^ = .59. Bonferroni corrected post hoc comparisons showed that participants spent a higher percentage of display time attending to alcohol than soda Brand, Packaging, and Glass cues (all *p*s < .001). The opposite was found for Portrayal cues, where participants spent a higher percentage of display time attending to Portrayal cues in soda adverts compared to alcohol adverts (*p* < .001).

#### Alcohol consumption

On average, participants consumed similar amounts of cider (*M* = 158.31, *SD* = 117.31) and cola (*M* = 156.37, *SD* = 100.65, paired samples *t* test *t*(61) = .16, *p* = .88).

In an initial analysis, we used a stepwise linear regression (backward elimination procedure) with participant characteristics (age, gender, AUDIT scores, weekly alcohol consumption, and motivation to reduce drinking) and attention to the responsible drinking statement, alcohol cues (sum of all individual alcohol cues), and soda cues (sum of all individual soda cues) as predictors of alcohol consumption. As shown in Table 4, age (β = .27, *t*(54) = 2.24, *p* = .03) and gender (β = −.27, *t*(54) = 2.24, *p* = .03) significantly predicted alcohol consumption: Male participants and older participants consumed more alcohol during the taste test. The other participant characteristics did not significantly predict alcohol consumption. Most importantly, attention to the responsibility statements, alcohol cues, or soda cues did not significantly predict alcohol consumption.[Table-anchor tbl4]

In order to test our second hypothesis that individual differences in attention to different types of alcohol cues would predict alcohol consumption, we conducted a second stepwise linear regression with backward elimination. We included participant characteristics, attention to the responsible drinking statements, and attention to the 4 different types of alcohol and soda cues (packaging, glass, brand presence, and sipping portrayal) as predictors of alcohol consumption. Similarly to the general model, age and gender were significant predictors of alcohol consumption; see Table 5. Regarding the attention variables, only attention to alcohol portrayal emerged as a significant predictor (β = .25, *t*(54) = 2.09, *p* = .04): Increased attention to the portrayal of alcohol consumption was predictive of increased alcohol consumption during the taste test.[Table-anchor tbl5]

#### Awareness of Drinkaware website

The majority of participants (91.9%, *n* = 57) correctly identified drinkaware.co.uk as the website displayed in alcohol advertising. Fifty of those (87.7%) reported being aware of the website before taking part in the study.

### Discussion

We measured alcohol consumers’ attention to responsible drinking statements and different types of alcohol cues in alcohol advertisements, and investigated how this was associated with their subsequent ad libitum alcohol consumption in a laboratory setting. Results showed that attention to the responsible drinking statements or general alcohol cues did not significantly predict alcohol consumption. However, analysis separated by alcohol cue type (alcohol packaging, alcohol drinks in a glass, portrayal of alcohol consumption, and brand logos) revealed that attention to the portrayal of alcohol consumption in adverts significantly predicted subsequent alcohol consumption: Participants who attended to alcohol portrayal longer, drank more alcohol during the taste test. There was no evidence that attention to any of the other alcohol cues predicted alcohol consumption. Additionally, we found that participants paid minimal attention to the responsible drinking statement in alcohol advertisements (∼1% of total display time), but most were still aware that the message referred to the Drinkaware website (91.9%).

Our findings are in line with previous research that showed that, when watching a movie, participants were more likely to drink alcohol in close temporal proximity to actors consuming alcohol, than at times when actors were not drinking alcohol (Koordeman, Kuntsche, et al., 2011), which accounted for increased total alcohol consumption in this group compared to another group of participants who did not see any alcohol portrayals (Koordeman, Anschutz, van Baaren, & Engels, 2011). However, a meta-analysis showed no overall effect of alcohol portrayal on immediate alcohol consumption (Stautz et al., 2016), possibly due to a lack of statistical power. The findings by Koordeman, Kuntsche, et al. (2011) might be understood in the context of social mimicry effects on alcohol consumption. For example, Larsen, Engels, Souren, Granic, and Overbeek (2010) showed that participants were more likely to consume alcohol in close temporal proximity to their drinking partner. Therefore, increased attention to alcohol portrayal in advertising may affect alcohol consumption by increasing mimicry. However, there has been no research on the relation between visual attention and social mimicry, so it is unclear whether greater attention also results in greater mimicry. Future research should investigate whether advertisements that portray alcohol consumption increase alcohol consumption to a greater extent than other alcohol advertisements. Additionally, it should be studied whether participants also mimic sipping behavior in advertisements, as they do with sipping behavior in films.

## General Discussion

In the studies presented here we measured visual attention to alcohol and responsible drinking statements in alcohol advertising and public health campaigns and investigated how this related to drinking intentions and drinking behavior in the lab. Both studies demonstrated that alcohol cues in alcohol adverts attract more attention than responsible drinking statements, even in a branded advertisement with a focus on responsible drinking. This finding is line with previous research. Kersbergen and Field (2017) and Thomsen and Fulton (2007) demonstrated that little attention is paid to responsible drinking statements on alcohol packaging and in print alcohol advertising, respectively. However, both studies found that participants paid some attention to the messages (∼7% of total viewing time in both studies), which is in contrast to findings from Study 2 that demonstrated that participants paid minimal attention to the responsibility statement if it was embedded in alcohol advertising (0.19 s over the course of 8 alcohol adverts; 0.65% of display duration). However, findings from Study 1 demonstrated that responsible drinking messages attracted more attention if they were embedded in a public health campaign or an alcohol advertisement that emphasized responsible drinking (43% and 7% of display duration, respectively). It is possible that responsible drinking statements are, by design, more visually salient in public health campaigns/adverts that emphasize responsible drinking and that this accounts for the increase in attention. Despite the lack of attention to the responsible drinking statement, message awareness in Study 2 was high. Therefore, it is likely that participants ignored the message (which was the same in each alcohol advert), because it did not provide them with any additional information. Additionally, in contrast to print advertising and packaging, imagery in TV advertising is constantly moving. Thus, participants need to actively prioritize attention to the cues they are interested in, because they are only displayed for a limited amount of time, whereas there are no time constraints when viewing print advertising or packaging.

We found no evidence that attention to responsible drinking statements or alcohol cues in general predicted drinking intentions or alcohol consumption in the lab. However, in Study 2 we demonstrated that visual attention to portrayals of alcohol consumption predicted ad lib alcohol consumption. Future research should investigate if removal of portrayals of alcohol consumption from alcohol advertising and public health campaigns moderates their influence on drinking intentions and behavior.

These studies had some limitations. First, we did not use a nonalcohol advert as a control condition in Study 1. Therefore, we do not know whether the public health campaign and alcohol advert with a focus on responsible drinking increased participants’ intentions to drink to the same extent as the alcohol advert, or whether none of the videos affected participants’ drinking intentions. Additionally, it was not possible to match the adverts in the video conditions on number, size and duration of alcohol cues and responsible drinking statements. Ideally, the adverts should show the same amount of alcohol/responsible drinking cues and only differ in the persuasive message, in order to make a fair comparison. Second, Study 2 was an observational within-subjects study in which all participants saw the same advertisements, so we cannot draw any conclusions regarding the effect of exposure to (specific) alcohol advertisements on alcohol consumption. Third, we used a limited number of advertisements and public health campaigns in both studies, which opens up the possibility that the adverts that we used were not representative of other alcohol adverts or public health campaigns. This is especially important in Study 1, as we only exposed participants to one advert/public health campaign per condition. It is therefore possible that the specific (non-)branded public health campaigns that we used were ineffective and that other adverts/campaigns would have resulted in significant differences in drinking intentions. The videos that we used in Study 1 did not portray all subtypes of alcohol cues (e.g., the public health campaign did not depict any specific alcohol brands, and the conventional alcohol advert did not portray people drinking), therefore we were unable to investigate the relationship between attention to all different subtypes of alcohol-related cues and alcohol-related outcomes in this study. Future research should investigate variability in public health campaigns and how specific themes and cue types affect drinking intentions. Fourth, because the literature on attention to different alcohol cue types was limited to still, pictorial stimuli, we categorized alcohol cues based on visual differences in product presentation. The categories were mutually exclusive and all display occasions of the alcohol product and responsible drinking statements were accounted for in one of the categories. However, it is possible that a different classification of alcohol cues (e.g., based on implied alcohol outcomes) would result in different findings. Fifth, in both studies we assessed individual differences such as recent alcohol consumption and motivation to reduce drinking after (rather than before) participants had been exposed to the alcohol-related adverts/public health campaign. Therefore, it is possible that participants’ responses to the questionnaires were affected by the videos that they had seen. However, in Study 1, we found no significant differences between groups on these variables, suggesting that exposure to the different videos did not robustly influence these variables. Finally, both male and female participants took part in Study 2 but we did not include female participants in Study 1 because we considered men to be the target audience for the advertisements that were used in Study 1 (whereas the advertisements used in Study 2 appeared to be aimed at a broader range of alcohol consumers, both men and women). This means that we cannot directly compare the findings from studies 1 and 2.

Our studies also had strengths. In Study 1, we used traditional alcohol advertisements as a control in order to directly compare the effect of ambiguous alcohol advertising (alcohol advert that emphasizes responsible drinking) to traditional advertising. In Study 2, we used advertisements for a variety of alcoholic drink types (cider, beer, and spirits) and brands, so we could capture attention to a range of alcohol cues. We used soda advertisements as control stimuli. This allows us to conclude that the association between attention to portrayal and alcohol consumption is specific to alcohol advertisements and not due to viewing actors drinking any type of beverage. In addition, in both studies we defined attention as a percentage of total cue display time, which allowed us to control for differences in display time between the alcohol/soda cues and responsibility statements.

To conclude, these studies demonstrated that responsible drinking statements in alcohol advertising attracted limited attention, but when viewing a public health campaign that was not associated with alcohol brands, participants attended more to the responsible drinking statements than to alcohol cues. However, individual differences in attention to responsible drinking statements did not predict drinking intentions or alcohol consumption in the laboratory. Out of all the alcohol cues, only attention to alcohol portrayal predicted ad lib alcohol consumption, but it did not predict drinking intentions. Future research should investigate how responsible drinking statements can be improved to attract more attention and prompt participants to intend to drink less or actually drink less alcohol. Additionally, it is important to study whether removal of alcohol portrayal from alcohol advertising and public health campaigns would affect drinking behavior.

## Supplementary Material

10.1037/adb0000284.supp

## Figures and Tables

**Table 1 tbl1:** Participant Demographics (Studies 1 and 2)

	Study 1				Study 2		
Variable	Heineken responsibility (*n* = 30) *M* (*SD*)	Heineken (*n* = 30) *M* (*SD*)	Drinkaware (*n* = 30) *M* (*SD*)	Total (*N* = 90) *M* (*SD*)	Males (*n* = 17) *M* (*SD*)	Females (*n* = 45) *M* (*SD*)	Total (*N* = 62) *M* (*SD*)
Age	24.27 (7.22)	21.37 (4.21)	24.00 (9.25)	23.21 (7.24)	24.88 (7.21)	22.87 (6.87)	23.42 (6.96)
AUDIT	8.57 (4.79)	10.27 (5.66)	11.03 (4.48)	9.96 (5.05)	14.59 (5.43)	11.47 (4.62)	12.32 (5.01)
Recent alcohol consumption (last 14 days)	27.65 (23.04)	41.67 (37.82)	47.37 (32.30)	38.89 (32.38)	34.56 (15.88)	21.48 (10.67)	25.06 (13.52)
Motivation to reduce drinking	1.75 (3.79)	1.08 (3.12)	1.53 (2.98)	1.45 (3.29)	3.97 (2.80)	1.13 (2.38)	1.91 (2.79)

**Table 2 tbl2:** Study 1: Advert/Video Characteristics

Variable	Drinkaware	Heineken responsibility	Heineken
Duration (s)	37	60	91
Display alcohol cues (% of duration)	12.43	13.18	12.94
Display responsible drinking statements (% of duration)	30.27	1.88	N/A
Number of alcohol cues	4	6	6
Number of responsible drinking statements	3	1	N/A
Size alcohol cues (cm^2^ × s) as a percentage of total display size	3.84	.75	.80
Size responsible drinking statements (cm^2^ × s) as a percentage of total display size	12.98	.04	N/A

**Table 3 tbl3:** Study 1: The Effect of Advertising Condition on Three Measures of Drinking Intentions

Measure of drinking intentions	Advertising condition		
	Heineken responsibility *M* (*SD*)	Heineken *M* (*SD*)	Drinkaware *M* (*SD*)
Next drinking occasion (UK units)	15.99 (21.14)	18.35 (19.71)	16.49 (18.91)
Next week (UK units)	13.23 (9.76)	15.23 (15.07)	17.60 (13.64)
Binge drinking intentions	3.43 (2.27)	3.93 (2.50)	4.71 (2.27)

**Table 4 tbl4:** Study 2: Stepwise Linear Regression Analysis (Backward Elimination) With Age, Gender, AUDIT Scores, Weekly Alcohol Consumption, Motivation to Reduce Drinking, and Attention to Drinkaware Messages, Alcohol Cues, and Soda Cues as Predictors of Ad-Lib Alcohol Consumption

Variable	Alcohol consumption
Age (β)	.28*
Gender (β)	−.29*
*R*^2^	.18
*F*(2, 55)	5.96**
Excluded variables	
AUDIT (β)	.05
Weekly alcohol consumption (β)	−.01
Motivation to reduce drinking (β)	.04
Attention to drinkaware message (β)	.06
Attention to alcohol cues (β)	.12
Attention to soda cues (β)	.10
* *p* < .05. ** *p* < .10.	

**Table 5 tbl5:** Study 2: Stepwise Linear Regression Analysis (Backward Elimination) With Age, Gender, AUDIT Scores, Weekly Alcohol Consumption, Motivation to Reduce Drinking, and Attention to Alcohol and Soda Cues (Drinkaware, Bottle, Brand, Glass, and Portrayal) as Predictors of Ad-Lib Alcohol Consumption

Variable	Alcohol consumption
Age (β)	.27*
Gender (β)	−.27*
Attention to alcohol portrayal (β)	.25*
*R*^2^	.24
*F*(3, 54)	5.67**
Excluded variables	
AUDIT (β)	.03
Weekly alcohol consumption (β)	.01
Motivation to reduce drinking (β)	.06
Attention to alcohol packaging (β)	.01
Attention to alcohol glass (β)	−.15
Attention to alcohol brand (β)	−.11
Attention to drinkaware message (β)	−.02
Attention to soda portrayal (β)	.01
Attention to soda packaging (β)	−.09
Attention to soda glass (β)	−.15
Attention to soda brand (β)	−.22
* *p* < .05. ** *p* < .10.	

## Footnotes

1 Including all participants in analysis, or using more stringent exclusion criteria based on outlier analysis, did not affect the pattern of results.

2 Including all participants in analysis, or using more stringent exclusion criteria based on outlier analysis, did not affect the pattern of results.

3 Attention to the responsible drinking statement did not differ over successive trials; see supplementary materials.
