# Supplementary material for: Visual Attention to Alcohol Cues and Responsible Drinking Statements Within Alcohol Advertisements and Public Health Campaigns: Relationships With Drinking Intentions and Alcohol Consumption in the Laboratory
Source: Psychol Addict Behav. 2017 May 11;31(4):435–46. doi: 10.1037/adb0000284 (PMC5467671; doi:10.1037/adb0000284)
Supplement: Supplementary file 1 [file ze6999173096so1.docx]

**Supplemental Materials**

**Visual Attention to Alcohol Cues and Responsible Drinking Statements Within Alcohol Advertisements and Public Health Campaigns: Relationships With Drinking Intentions and Alcohol Consumption in the Laboratory**

**by I. Kersbergen & M. Field, 2017, *Psychology of Addictive Behaviors***

**http://dx.doi.org/10.1037/adb0000284**

**Coding of alcohol cues (studies 1 and 2)**

In both studies, alcohol cues were categorised as those depicting: 1) Portrayal: occasions where a person taking a sip of the advertised product was displayed on screen; 2) Packaging: occasions where a branded bottle or can of the advertised product was displayed (excluding occasions that fit under Portrayal); 3) Glass: occasions where the advertised product was displayed in a glass (excluding occasions that fit under Portrayal); 4) Logo: occasions where the brand logo was displayed separately from the product. To ensure that each cue was only categorized into one type of cue, packaging and glass cues that were seen as part of portrayal cues were only coded as portrayal cues and not coded as packaging/glass cues.

In study 1, only the Drinkaware video displayed any portrayal cues, all of which involved alcohol displayed in a glass. If the portrayal cues had instead been coded as glass cues, they would have accounted for 70% of alcohol glass display time. In study 2, all soda portrayal cues involved product packaging. 66% of alcohol portrayal display time involved product packaging, the remaining 34% involved products displayed in glasses. If the portrayal cues had instead been coded as packaging/glass cues, they would have accounted for 2.6% of alcohol glass display time, 8.4% of alcohol packaging display time and 15.0% of soda packaging display time.

**Attention to responsible drinking statements over time (study 2)**

We conducted multilevel modelling to analyse attention to responsible drinking statements as a function of presentation order. Data were organised with advertisements (n = 8) nested within participants (n = 58). The multilevel model that included order of presentation as a predictor (*b* < .001, SE < .001) was not a significant improvement on an intercept-only model (χ^2^(1) = 0.001, p = .97). This indicates that attention to responsible drinking statements did not depend on whether the statement was presented in the first advert they viewed or a later advert.


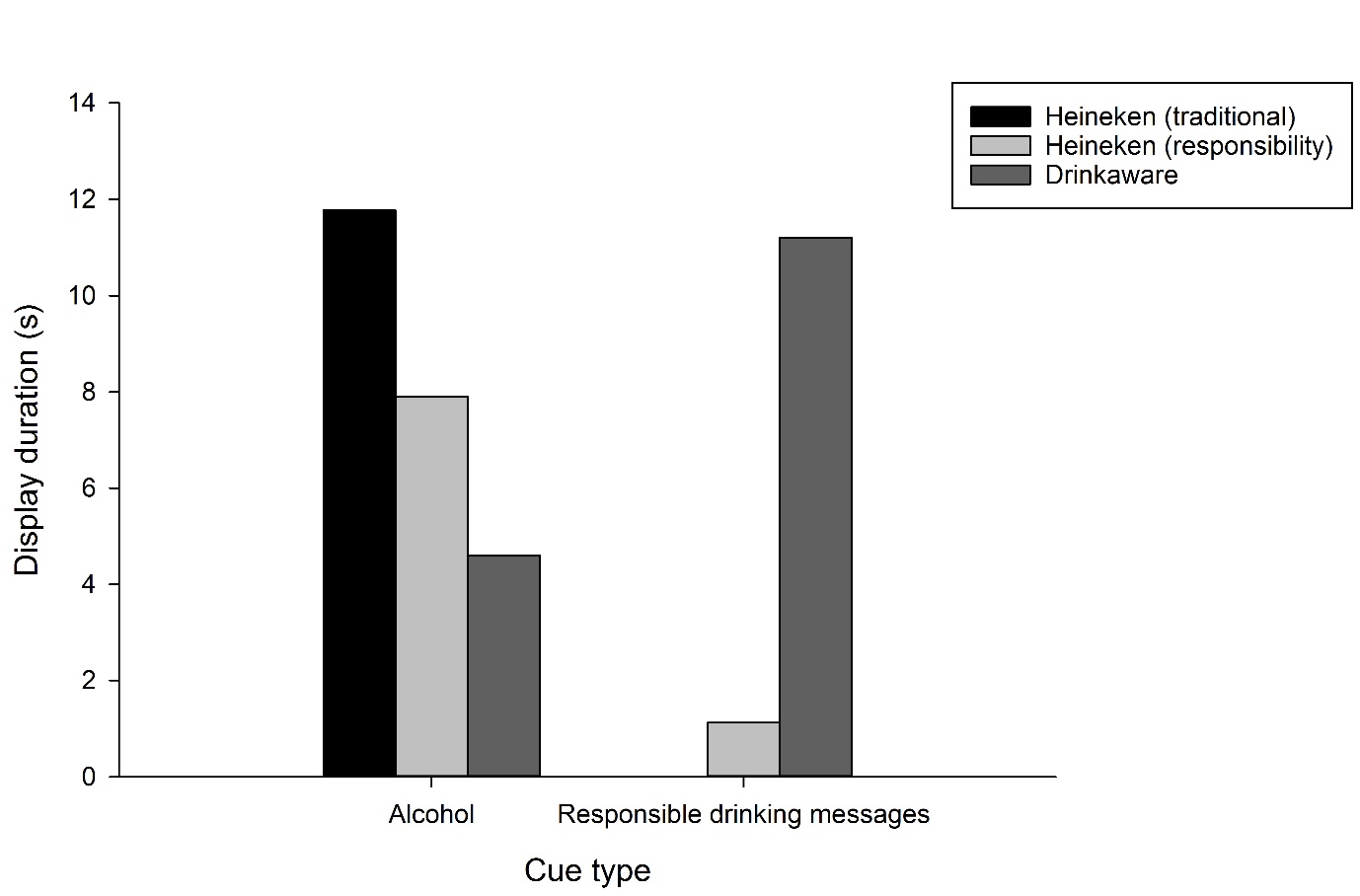


*Figure S1.* Study 1. Total display duration of alcohol cues and responsible drinking statements in the different advertising conditions. Note: Heineken advert did not display any responsible drinking statements.


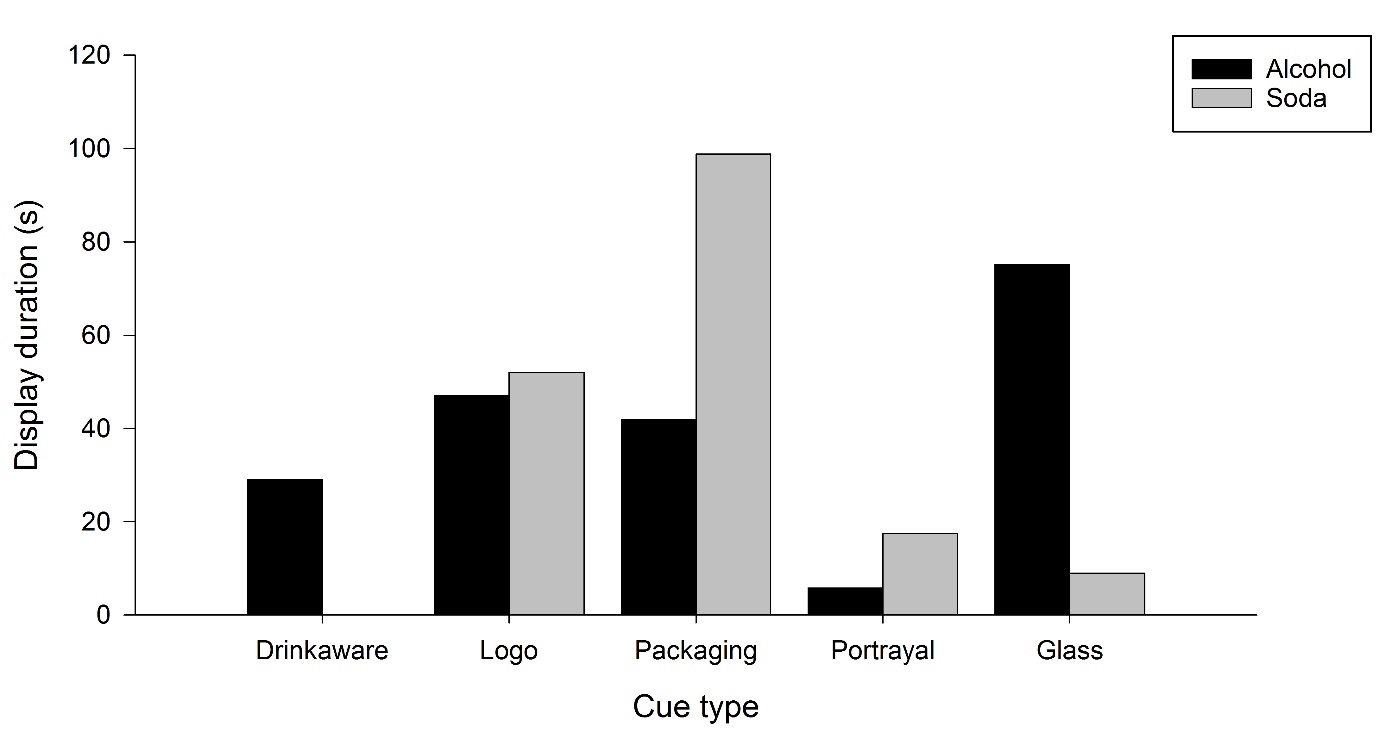


*Figure S2.* Total display duration in seconds of responsible drinking statements (Drinkaware website; alcohol adverts only), and Brand, Packaging, Portrayal, and Glass cues in alcohol and soda adverts.

| **Table S1.** Study 1. Correlations between attention to alcohol, health and various alcohol marketing cues (logos, packaging, glasses, portrayal) and three measures of drinking intentions, across the three advertising conditions, controlling for participant characteristics (age, AUDIT scores, weekly alcohol consumption and motivation to reduce drinking). | | | | |
| --- | --- | --- | --- | --- |
| Condition | Attention variable | Next drinking occasion (*r*) | Next week (*r*) | Binge drinking intentions (*r*) |
| Heineken responsibility (n = 28) | Attention to alcohol cues | .09 | -.17 | -.04 |
|  | Attention to health cues | .15 | -.12 | .-.07 |
|  | Attention to alcohol packaging | -.02 | -.27 | -.21 |
|  | Attention to alcohol logos | .14 | .05 | .16 |
|  | Attention to alcohol glasses | N/A | N/A | N/A |
|  | Attention to alcohol portrayal | N/A | N/A | N/A |
| Heineken (n = 26) | Attention to alcohol cues | .29 | -.05 | -.27 |
|  | Attention to health cues | N/A | N/A | N/A |
|  | Attention to alcohol packaging | .33 | -.09 | -.13 |
|  | Attention to alcohol logos | .18 | -.01 | -.31 |
|  | Attention to alcohol glasses | N/A | N/A | N/A |
|  | Attention to alcohol portrayal | N/A | N/A | N/A |
| Drinkaware (n = 25) | Attention to alcohol cues | -.18 | -.15 | .07 |
|  | Attention to health cues | .04 | .03 | -.22 |
|  | Attention to alcohol packaging | N/A | N/A | N/A |
|  | Attention to alcohol logos | N/A | N/A | N/A |
|  | Attention to alcohol glasses | -.09 | -.23 | -.04 |
|  | Attention to alcohol portrayal | -.23 | -.06 | .15 |
| Note: ^+^*p* < .10 | | | | |
